# CisBP-RNA: a web resource for eukaryotic RNA-binding proteins and their motifs

**DOI:** 10.1093/nar/gkaf1081

**Published:** 2025-10-29

**Authors:** Diego A Rosado-Tristani, Mihai Albu, Xiaoting Chen, Alexander Sasse, Kaitlin U Laverty, Debashish Ray, Cyrus L Tam, Kevin Ernst, Lucinda P Lawson, Quaid D Morris, Timothy R Hughes, Matthew T Weirauch

**Affiliations:** Division of Allergy and Immunology, Cincinnati Children’s Hospital Medical Center, Cincinnati, OH 45229, United States; Donnelly Centre, University of Toronto, Toronto, ON M5S3E1, Canada; Center for Autoimmune Genomics and Etiology, Cincinnati Children’s Hospital Medical Center, Cincinnati, OH 45229, United States; Center for Molecular Biology Heidelberg (ZMBH), Heidelberg University, 69049, Germany; Sloan Kettering Institute, Memorial Sloan Kettering Cancer Center, New York, NY 10065, United States; Donnelly Centre, University of Toronto, Toronto, ON M5S3E1, Canada; Graduate Program in Computational Biology and Medicine, Weill Cornell Graduate School, New York, NY 10065, United States; Center for Autoimmune Genomics and Etiology, Cincinnati Children’s Hospital Medical Center, Cincinnati, OH 45229, United States; Center for Autoimmune Genomics and Etiology, Cincinnati Children’s Hospital Medical Center, Cincinnati, OH 45229, United States; Sloan Kettering Institute, Memorial Sloan Kettering Cancer Center, New York, NY 10065, United States; Donnelly Centre, University of Toronto, Toronto, ON M5S3E1, Canada; Department of Molecular Genetics, University of Toronto, Toronto, ON M5S3E1, Canada; Division of Allergy and Immunology, Cincinnati Children’s Hospital Medical Center, Cincinnati, OH 45229, United States; Center for Autoimmune Genomics and Etiology, Cincinnati Children’s Hospital Medical Center, Cincinnati, OH 45229, United States; Division of Developmental Biology, Cincinnati Children’s Hospital Medical Center, Cincinnati, OH 45229, United States; Division of Biomedical Informatics, Cincinnati Children’s Hospital Medical Center, Cincinnati, OH 45229, United States; Division of Human Genetics, Cincinnati Children’s Hospital Medical Center, Cincinnati, OH 45229, United States; Department of Pediatrics, University of Cincinnati College of Medicine, Cincinnati, OH 45229, United States

## Abstract

RNA-binding proteins (RBPs) are key mediators of post-transcriptional gene regulation, including splicing, transport, stability, and other facets of RNA metabolism. Many RBPs exert their function through sequence-specific protein–RNA interactions. RBP RNA-binding specificity models, or motifs, are thus essential for understanding post-transcriptional gene regulatory mechanisms. Here, we present CisBP-RNA (Catalog of inferred sequence Binding Preferences of RNA-binding proteins), a freely available web-based database that provides centralized access to eukaryotic RBP motif data. A key feature of CisBP-RNA is the availability of both experimentally determined motifs and motifs that are computationally predicted via our homology-based approaches. The current version of CisBP-RNA catalogs >148 000 sequence-specific RBPs across 690 eukaryotic species. Motifs are currently available for >34 000 of these RBPs. Motif data are available for download in a variety of formats for downstream computational analyses. In addition, CisBP-RNA provides user-friendly web-hosted tools to scan for predicted RBP binding sites, predicts motifs for a protein of interest, and compares a motif to motifs contained in the database. The CisBP-RNA database can be accessed through www.cisbp.org/rna/.

## Graphical Abstract

**Graphical Abstract:**
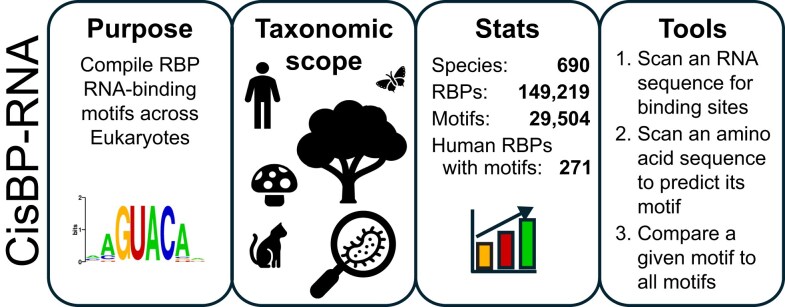

## Introduction

Gene regulation is the process controlling the timing, location, and amount in which genes are expressed. During transcription, gene regulation is primarily controlled by sequence-specific DNA-binding proteins called transcription factors (TFs). Genes are also regulated post-transcriptionally, largely through the actions of RNA-binding proteins (RBPs) [1]. RBPs control a range of messenger RNA (mRNA)-based processes, including splicing, editing, stability, and localization. RBPs also play important roles in many human diseases. For example, altered RBP function is thought to be an important mechanism in multiple types of cancer [2–5]. Much like TFs, RBP–nucleic acid interactions can be highly sequence-specific [6]. To achieve this specificity, most known RBPs possess a modular architecture containing at least one RNA-binding domain (RBD), which is also used to assign RBPs into protein families [7]. Across eukaryotes, there are approximately a dozen well-characterized RBP families, with many likely yet to be discovered [8].

Identification of the *in vivo* mRNA-binding targets of RBPs is a major goal in gene regulation research. Progress toward this goal has been made possible thanks to advances in experimental methods that interrogate RNA–protein interactions, such as crosslinking and immunoprecipitation (CLIP) coupled with sequencing. In a CLIP-seq experiment, RNA–protein complexes are crosslinked, enriched through immunoprecipitation, isolated, sequenced, and then mapped to the transcriptome to identify RBP binding locations [9]. However, numerous challenges limit motif discovery using this strategy, including (i) difficulties obtaining sufficient material for experiments [10], (ii) a reliance on CLIP-grade antibodies, which are not commercially available for most RBPs, (iii) the influence of context-specific factors (e.g. RNA structure and other RBPs can influence binding site selection), and (iv) differential abundance and localization of both transcripts and RBPs within cells.

Methods performed *in vitro* can overcome these challenges while also introducing new limitations. For example, RNAcompete [9] and HTR-SELEX (high throughput, RNA-based systematic evolution of ligands by exponential enrichment) [11] both measure RBP binding preferences using a large pool of synthetic RNA sequences. RNAcompete experiments typically contain ∼240 000 30–41-base sequences designed to contain every possible 7-base sequence at least 155 times in varying sequence contexts. HTR-SELEX oligo pools typically contain ∼10^12^ randomly generated 40-base sequences. Other recently developed methods can assess both sequence and structural binding specificity (RNA Bind-n-Seq) [12] or measure the RNA-binding specificity of protein complexes [13]. In all of these approaches, bound oligos are used to identify short, recurring sequence patterns that can be used to create a motif. While these methods are highly effective at establishing innate RNA-binding specificity, factors beyond specificity guide RBP *in vivo* targeting, such as RNA secondary structure and interactions with other cellular proteins.

**Figure 1.**
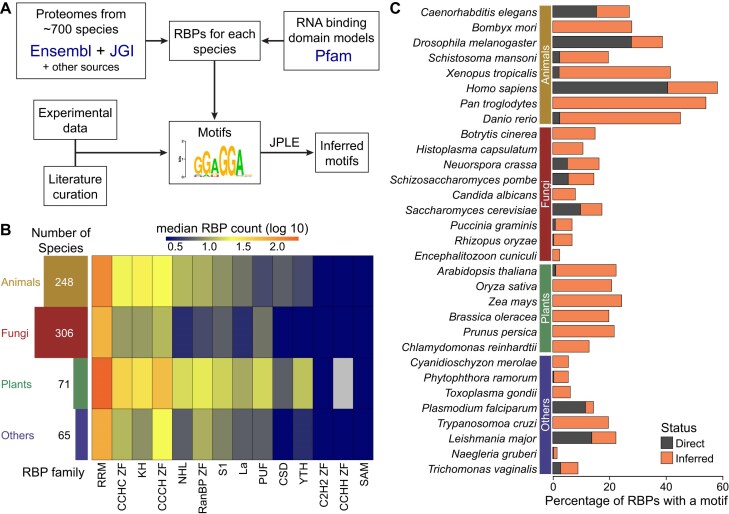
Summary of the CisBP-RNA (Catalog of inferred sequence Binding Preferences of RNA-binding proteins) resource. (**A**) Workflow summarizing the underlying CisBP-RNA methodology. Public data, new experimental data, and advanced computational workflows combine to provide a platform for the exploration of RBPs and their motifs. (**B**) Species and RBP abundance across taxonomic clades. The left bar plot indicates the number of species in each clade. The heatmap shows the median RBP count for the given RBD family in the given clade, on a log 10 scale. (**C**) RBP motif counts across representative species. Blue bars on the *Y* axis indicate the percentage of RBPs with at least one directly determined motif. Green bars indicate the percent of RBPs lacking a direct motif with at least one inferred motif.

The term motif in the context of RBP binding specificities serves as a general descriptor for binding models. Motifs are not binary sequences of affinity (i.e. bound versus unbound), but instead quantitatively represent the specificity and relative strength of potential interactions between a given RBP and RNA based on RNA sequence. These models account for the fact that RBPs tolerate some sequence variability while still maintaining binding capacity, and that certain nucleotides at specific positions contribute more strongly to binding affinity than others. The primary representation of such motifs is the position frequency matrix (PFM). PFMs were initially developed to represent the frequency of each nucleotide at each position within a known binding site [14, 15]. A PFM is organized as an *m* × *n* matrix, in which *m* is the length of the binding motif and *n* represents the four possible ribonucleotides. Each row–column intersection represents the frequency for ribonucleotide *n* at position *m*, with these values often being visualized as motif “sequence logos” (Fig. 1A). These matrices can then be used to score new RNA sequences, identifying regions that closely match the motif and are likely to be potential binding sites for the RBP. In addition to PFMs, RNAcompete experiments are designed to systematically produce RBP binding specificity data in the form of *k-*mer scores, e.g *Z*-scores [16]. This simple model provides a “lookup table” that scores, for instance, each of the 16 382 possible 7-mers, from “AAAAAAA” to “UUUUUUU.”

Given the importance of RBPs, a handful of RBP motif databases have been created. One of the earliest, RBPDB, was published by our group in 2011, and features manually curated data for 1337 RBPs and 71 motifs across four model organisms: human, mouse, fly, and worm [17]. The ATtRACT database, published in 2016, contains 374 RBPs across 38 species and ∼4500 motifs, including motifs contained in RBPDB [18]. Our own efforts recently resulted in the EuPRI resource, which contains 174 new eukaryotic RNAcompete-derived motifs, along with a new method called "Joint Protein-Ligand Embedding" (JPLE) for inferring motifs across species [8]. These new data and methods have been incorporated into the CisBP-RNA system. Despite these efforts, many RBPs remain uncharacterized, and thousands of RNA-binding motifs have yet to be discovered.

Here, we present the CisBP-RNA. CisBP-RNA is a comprehensive database of sequence-specific RBPs and their motifs. Initially published over a decade ago [16], the current build of CisBP-RNA contains thousands of RBP motifs for nearly 700 eukaryotic organisms [8]. In total, 632 experimentally determined motifs obtained from 14 individual sources and our RBPDB resource [17] are provided, including RNAcompete and HTR-SELEX experiments performed by our group and others. We also provide homology-based computationally predicted motifs, based on our JPLE method [8]. This dual approach provides broad motif coverage, even for RBPs in nonmodel organisms where direct experimental data may be limited or nonexistent. As a result, CisBP-RNA serves as a powerful resource for researchers investigating post-transcriptional gene regulation, including in less-characterized species. The database’s cross-species scope also makes it especially valuable for studying evolutionarily conserved regulatory elements and for inferring functional relationships among RBPs across different lineages. A suite of tools is also provided to predict RNA-binding sites and identify RBPs and their motifs. CisBP-RNA is freely accessible at www.cisbp.org/rna, offering a user-friendly interface for both exploratory research and advanced downstream RBP analyses.

## Results

### Overview of the CisBP-RNA resource

CisBP-RNA contains information on RBPs and their motifs obtained from multiple sources, using a semiautomated computational workflow (Fig. 1A). First, eukaryotic proteomes are retrieved from a variety of sources, including JGI [19] and Ensembl [20]. Next, putative RBPs are identified in each proteome by scanning for predicted RBDs with HMMER [21] using profile hidden Markov models (pHMMs) obtained from the Pfam database [22] (Supplementary Table S1), which is now hosted within InterPro [23]. Starting with CisBP-RNA build 2.0, the workflow uses a version of the “RRM_1” pHMM model that has been slightly modified to include additional flanking amino acids that have been shown to be important for RRM binding specificity [8]. Next, RBPs are associated with experimentally established motif data though either manual curation of public datasets or via newly generated motifs obtained through RNAcompete or HTR-SELEX experiments. Finally, experimentally derived motifs are used to infer RBP binding specificities across and within species using either our JPLE algorithm (for the large RRM and KH families) [8] or RBD sequence identity (for all other families) [16]. Details are provided in [8]. In brief, JPLE leverages relationships between RBD protein sequence and RNA sequence specificity to predict RNA specificity from the amino acid sequences of RBDs. It employs a representation learning framework to impute RNAcompete (7-mer) binding profiles from protein peptide representations. In addition, JPLE also provides a confidence score for its predictions, and uses the joint representation learning to determine binding peptide importance scores, which are used to create a map of predicted RNA-contacting residues.

The current version of CisBP-RNA (2.0) catalogs RBPs in 690 eukaryotic species spread across all major clades (Fig. 1B). The underlying data are organized in a set of 25 MySQL relational tables, along with flat files in various formats for downloads. In total, the database currently contains >148 000 RBP, 1219 experimentally derived motifs, and 34 242 inferred motifs (Table 1). RBPs are organized into families based on their RBDs. The RRM family is the largest RBP family across eukaryotes, accounting for over half of the RBP repertoire in most species (Fig. 1B). Certain families show interesting lineage-specific patterns. For example, fungi have far fewer NHL-containing RBPs than the other clades. Collectively, our approach has substantially expanded motif coverage across eukaryotes, with hundreds of motifs available for many species, including model organisms such as *Danio rerio, Xenopus tropicalis, Mus musculus*, and *Arabidopsis thaliana* (Supplementary Table S2). In particular, CisBP-RNA currently contains 465 human RBPs. Forty-one percent of these RBPs have an experimentally determined motif, and an additional 18% have inferred motifs. For nonmodel organisms, most motifs are inferred. For example, among primates (e.g. chimpanzee), most motifs are inferred from human motifs (Fig. 1C and Supplementary Table S2). By providing >30 000 motifs for ∼700 species in a single user-friendly web interface, CisBP-RNA provides an unrivaled resource for the systematic study of eukaryotic post-transcriptional mechanisms.

**Table 1.** CisBP-RNA contents in the previous and current database builds

	Previous build	Current build
Species	289	690
RBPs	62 587	148 533
Experimental motifs	271	1219
RBPs with direct motifs	250	504
RBPs with inferred motifs	7806	34 242
Motif sources	2	15

### The CisBP-RNA website user experience

**Figure 2.**
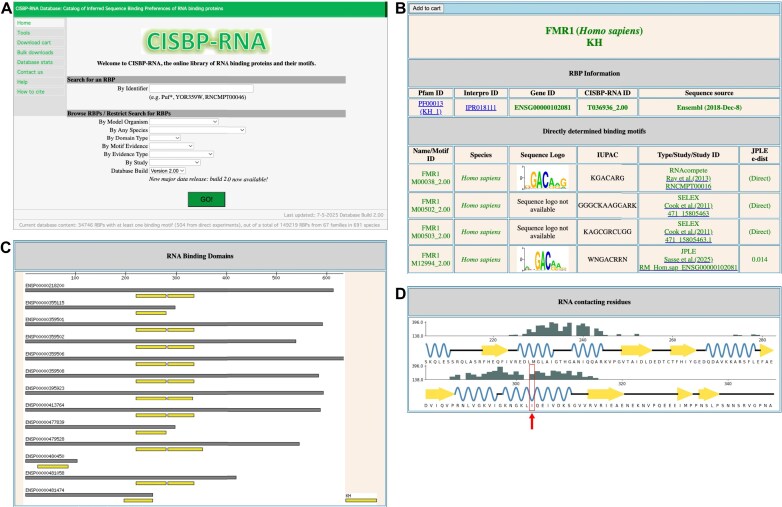
Navigating CisBP-RNA and accessing its data. (**A**) Users can access information quickly from the CisBP-RNA homepage, via a search bar, browsing filters, or direct links. (**B**) Each catalogued RBP has its own subpage, providing general information (e.g. RBP family, RBD ID, etc.), links to other databases, and direct and inferred motifs. (**C**) The RBP subpage also contains a section indicating the locations of RBDs within each protein isoform. (**D**) A JPLE-derived RNA contact map highlights the amino acid residues predicted to directly interact with RNA. In this example, the red arrow indicates Ile304 of the FMR protein, which has been experimentally shown to impact FMR RNA-binding activity.

The CisBP-RNA homepage is designed to provide easy access to RBP and motif information through a variety of search and selection panels (Fig. 2A). Users can either search for specific RBPs using the search bar or browse by species, domain type, or experiment type using drop-down filters. Each RBP has a dedicated subpage displaying key identifiers (Pfam or Interpro domain ID, Gene ID, CisBP-RNA ID, and sequence source), directly determined motifs with associated metadata, inferred motifs (by JPLE or amino acid identity), and relationships to other RBP family members based on RBD homology (Fig. 2B). Additionally, the RBP subpage also contains information on RBD locations for each protein isoform (Fig. 2C). Finally, information on predicted RNA-contacting residues is provided (Fig. 2D). Mutations affecting RNA-interacting residues can alter RNA-binding specificity and/or affinity. Thus, the JPLE-predicted residue contact maps can aid in identifying variants that might disrupt RBP binding. For example, a mutation in FMR1 (Ile304Asn) identified in a patient with Fragile X syndrome led to decreased FMR RNA-binding activity [24]. This position is predicted by JPLE to have a high likelihood of contacting RNA (Fig. 2D), demonstrating how the CisBP-RNA residue contact map can help users prioritize variants to study.

Multiple avenues are provided for data download on CisBP-RNA. Download types include RBP metadata, motifs (in the form of PFMs) and sequence logos, and RNAcompete-derived *k*-mer *E*-score and *Z*-score models. From individual RBP subpages, RBP and their motifs can be added to a cart, which can be used to download associated information for the subset of added RBPs. For broader data access, the Bulk Downloads section allows users to download entire datasets, from all data to useful slices of data, such as all human RBPs or all KH family motifs.

### CisBP-RNA tools and case studies

A long-standing challenge in computational biology is to “explain” gene expression patterns based on elements within regulatory sequences [25, 26]. CisBP-RNA helps to address this challenge by offering a suite of tools for exploring RBP function in a variety of settings. To illustrate the practical utility of CisBP-RNA for these purposes, we present these tools along with a case study to demonstrate how the CisBP-RNA resource can be used to address specific research topics.

#### Scanning RNA sequences for putative RBP binding sites

**Figure 3.**
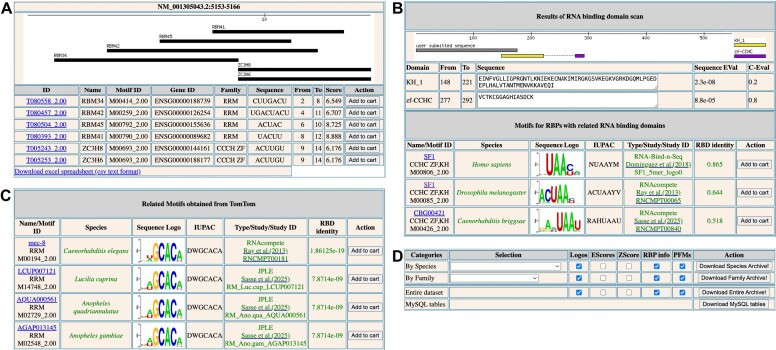
CisBP-RNA tools. (**A**) “RNA Scan” results page. The “RNA Scan” tool identifies putative RBP binding sites within an input RNA sequence, using three different scoring systems. (**B**) “Protein Scan” results page. The “Protein Scan” tool identifies predicted RBDs in an input protein sequence and predicts the motif that the protein will recognize based on homology to all proteins in the CisBP-RNA database. (**C**) “Motif Scan” results page. The “Motif Scan” tool identifies motifs in the database that are similar to the input motif. (**D**) The “Bulk Downloads” page provides data downloads in multiple formats and is easily adjustable to user needs (e.g. “All data,” “human data only,” “PFMs only,” etc.).

In order to predict potential post-transcriptional regulatory mechanisms, sequences of interest (e.g. regions proximal to alternative splicing events) are often computationally scanned for motif matches to potential RBP binding sites. To this end, CisBP-RNA includes the “RNA Scan” tool, which scans input RNA sequences for matches to motifs of interest. Motifs can be selected for a particular species, or for motifs contained in the user cart. Three scoring methods are included as options: log-likelihood ratio (the classic method for scanning for position weight matrix (PWM) matches) [27], energy based (a newer PWM-scanning method based on biophysical interaction estimates) [28], and 7-mer *E*-score based (a *k*-mer model specific to RNAcompete data that provides a “lookup table” of scores for all 16 382 possible 7 base sequences) [16]. Scans can be performed using default or user-specified cutoffs. Scan results provide a visual representation of the location of predicted binding sites within each input sequence, along with associated statistics and links to the RBP pages corresponding to the predicted binding sites (Fig. 3A).

#### Identifying putative RBDs and predicting motifs through analysis of protein sequences

Functional information on newly sequenced genomes is often sparse, making the identification of functional domains within protein sequences an important first step in deciphering protein functions. To this end, CisBP-RNA includes the “Protein Scan” tool, which detects putative RBDs within user-provided protein sequences and attempts to predict the corresponding motif(s) that would be recognized. This search is performed using pHMMs, which represent patterns and statistical properties of evolutionary related protein sequences [29]. Protein Scan performs this search using HMMER and a curated set of RBD HMM profiles obtained from the Pfam database [22]. The identified RBDs are then compared to all RBDs in the CisBP-RNA database to predict the corresponding RNA motif. As output, the tool provides all predicted RBDs contained within the user sequence, both in pictorial form and in table format, with associated statistics and links (Fig. 3B). If motif predictions are possible, a ranked list is provided, in descending order of likelihood (based on RBD % amino acid identity), along with links to the corresponding RBPs’ webpages.

#### Identifying related motifs through motif comparison

It is often useful to compare novel experimentally or computationally determined motifs to existing RBP motifs, in order to identify potential function via similarity. Further, for RBP motifs obtained from organisms not yet contained within CisBP-RNA, motif comparisons can provide insights into regulatory mechanism evolution. The “Motif Scan” tool facilitates such queries by comparing a user-provided motif against motifs contained in CisBP-RNA using the TomTom algorithm [30]. Multiple input formats are accepted including PFMs, IUPAC ambiguity codes, and multisequence alignments. As output, related motifs are provided, sorted in order of similarity, along with links to the associated RBPs (Fig. 3C).

#### Case study: identifying putative RBPs and their associated motifs for a newly sequenced organism

**Figure 4.**
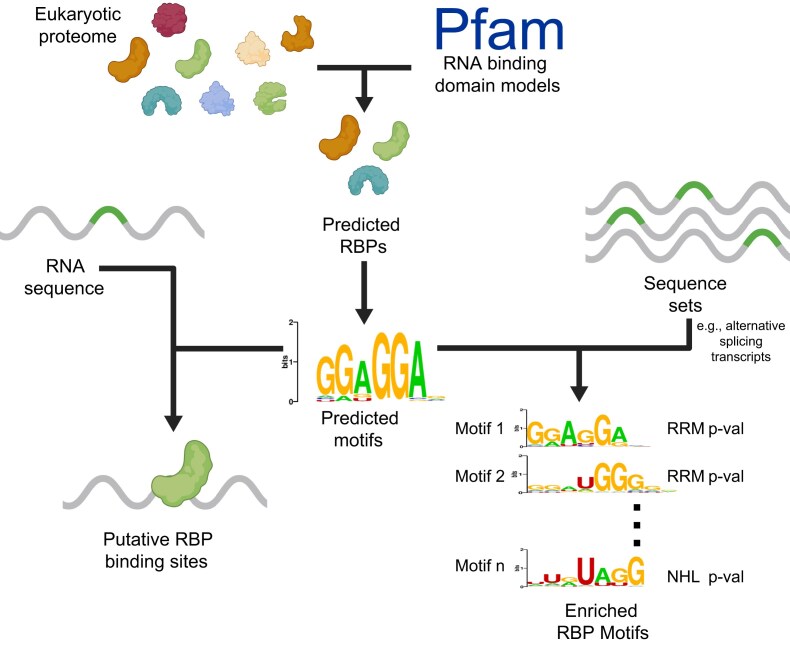
Case study: annotating a newly sequenced genome with predicted RBPs, motifs, and gene regulatory mechanisms. Data contained in CisBP-RNA can be readily used to annotate newly sequenced genomes. First, the proteome is scanned for putative RBPs (top). Next, motifs are predicted for these RBPs using the JPLE method (middle). The resulting motif set can then be used to scan RNA sequences for predicted RBP binding sites (bottom-left) or to perform motif enrichment analysis on sets of related RNA sequences (e.g. alternatively spliced transcripts; bottom-right). This figure was created in BioRender: Rosado, D. (2026) https://BioRender.com/58i90t2.

While each tool in CisBP-RNA can be used independently for simple queries, computationally intensive tasks such as analyzing a newly sequenced organism are best performed locally. CisBP-RNA supports such analyses by providing extensive data download capabilities. Here, we describe a general workflow that can be run locally on the command line in order to provide a comprehensive assessment of the RBPs encoded in the proteome of a newly sequenced species, along with their predicted motifs and predicted transcriptome binding sites (Fig. 4).

First, putative RBPs are identified in the genome assembly by using the HMMER tool and the 14 Pfam RBD pHMMs to scan the amino acid sequences derived from gene model predictions. Next, motifs are inferred by comparing the RBD sequence of each predicted RBP to CisBP-RNA sequence and motif data using JPLE and sequence identity. Finally, a motif scanning tool (e.g. MOODS [31]) is used to scan transcriptomes to predict potential RBP binding sites. RBP motif enrichment analysis (e.g. using HOMER [32]) can also be performed, if genes or gene regions of interest are available through RNA-seq (e.g. sets of alternatively spliced cassette exons), as in [33]. Likewise, enriched CLIP-seq peak sets can be identified within these regions using data from ENCODE [34] and the RELI algorithm [35], as in [36]. Upon completion of these steps, the newly sequenced genome will have (i) predicted RBPs, (ii) inferred motifs for these RBPs, (iii) predicted RBP binding sites in the form of motif matches within each gene transcript, and (iv) (optional) RBP-target predictions for gene sets of interest.

## Conclusions and future directions

Post-transcriptional gene regulation plays essential roles in many biological functions. RBPs are a critical component of these processes. Despite their functional importance, there are many aspects of RBPs that are not yet fully understood. In this work, we present CisBP-RNA, a comprehensive resource for eukaryotic RBPs and their motifs. CisBP-RNA is designed to facilitate both exploratory and hypothesis-driven research into post-transcriptional gene regulation, supporting a wide range of research applications, including RBP binding site prediction and RBP motif inference. The current build of CisBP-RNA contains 690 species spanning >148 000 RBPs, of which nearly 34 000 have one or more associated motifs. The broad scope of CisBP-RNA facilitates cross-species motif and binding site comparisons, enabling the interrogation of post-transcriptional gene regulation even in newly sequenced genomes. Through its comprehensive data integration, user-friendly interface and tools, and powerful data download interface, CisBP-RNA greatly enhances accessibility and interpretability in RBP research.

CisBP-RNA also has a “sister database” of TFs and their motifs: CisBP (www.cisbp.org/dna) [37]. CisBP provides similar data types and similar functionality for the study of transcriptional regulation. Together, CisBP and CisBP-RNA provide powerful resources for the study of gene regulation.

Ongoing efforts aim to expand the CisBP-RNA to include newly characterized RBP motifs, build better RBP binding models, and improve the online interface. The characterization of new motifs is central to the advancement of this field, as most eukaryotic RBPs still have unknown motifs. New data, when available, can be used to augment motif inferences across multiple species, including those that do not yet have experimentally determined motifs. The creation of more accurate models of RBP–RNA interactions is also crucial for advancing the field. Future CisBP-RNA builds aim to include motif models that incorporate elements such as RNA secondary structures (e.g. PRIESSTESS [38]) and chemical modifications (e.g. *N*^6^-methyladenosine/m^6^A [39]). Another priority is to update the CisBP-RNA user interface to enhance usability. The original website was conceived in the early 2010s, using then-current techniques for building dynamic web applications. Although the current implementation is a testament to the durability of this design, new technological developments continue to drive ongoing development. Updates that we are currently implementing include adding more intuitive searching and filtering queries and developing an API to facilitate command-line data queries and the integration of datasets into third-party analysis pipelines. These developments will ensure that CisBP-RNA remains a dynamic and evolving resource at the forefront of RBP research.

For over a decade, CisBP-RNA has provided access to eukaryotic RBPs, their motifs, and tools supporting post-transcriptional gene regulation research. During this time, it has become an invaluable and unique web resource. As the RBP research community continues to produce new data and methods, CisBP-RNA will likewise expand in contents, scope, and impact.

## Supplementary Material

gkaf1081_Supplemental_File

## Data Availability

CisBP-RNA can be accessed through www.cisbp.org/rna. The version of the JPLE corresponding to this publication is available at https://zenodo.org/records/17246481. Subsequent updated versions of this codebase can be found in Github at https://github.com/morrislab/RBPbinding.
